# Laboratory diagnostics in personalised medicine - 36th Symposium of the Croatian society of medical biochemistry and laboratory medicine

**DOI:** 10.11613/BM.2026.030501

**Published:** 2026-08-10

**Authors:** Dunja Rogić, Livija Šimičević, Tamara Žigman, Ivan Bambir, Andrea Vukić Dugac, Duška Tješić-Drinković, Ksenija Fumić, Ivan Lehman, Ivana Rako, Marina Popović, Lana Ganoci, Maja Živković, Alma Mihaljević-Peleš

**Affiliations:** 1 Faculty of Pharmacy and Biochemistry, Zagreb, Croatia; 2 Department of Laboratory Diagnostics, University Hospital Centre Zagreb, Zagreb, Croatia; 3 School of Medicine, University of Zagreb, Zagreb, Croatia; 4 Department of Pediatrics, University Hospital Centre Zagreb, Zagreb, Croatia; 5 Cystic Fibrosis Centre for Children and Adults, University Hospital Centre Zagreb, Zagreb, Croatia; 6 Clinic for respiratory diseases Jordanovac, University Hospital Centre Zagreb, Croatia; 7 Department of Oncology, University Hospital Centre Zagreb, Zagreb, Croatia; 8 Department of Psychiatry and Psychological Medicine, Zagreb University Hospital Centre, Zagreb, Croatia

**Keywords:** precision medicine, genetic diseases, neoplastic syndromes, pharmacogenomics, newborn screening

## Abstract

Personalised medicine has become a central paradigm in modern healthcare, aiming to tailor prevention, diagnosis, and therapy to each patient’s unique molecular and clinical profile. This paper provides a comprehensive overview of the lectures presented at the 36th annual Symposium of the Croatian society of medical biochemistry and laboratory medicine. It highlights key topics including the role of disease-causing variants in hereditary disorders, the development of cystic fibrosis transmembrane conductance regulator (CFTR) modulators, the evolution of newborn screening programs and spinal muscular atrophy diagnostics, emphasizing early detection and timely initiation of therapy, advances in cancer genetics, pharmacogenetics, and the translation of other laboratory-based innovations into clinical practice. Altogether, these advances reaffirm the central role of laboratory diagnostics as the cornerstone of personalised medicine, bridging genetic discovery with clinical translation and transforming modern healthcare into a predictive, preventive, and truly patient-centred discipline.

## Graphical abstract

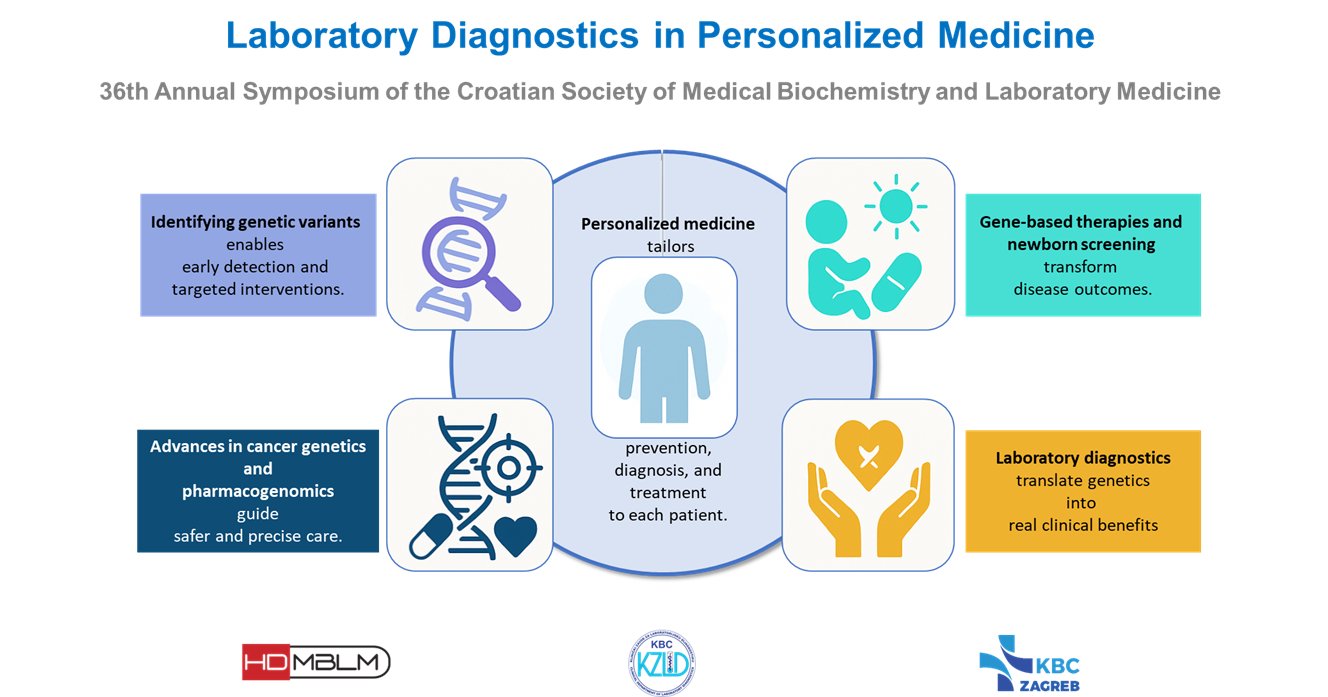

## Highlights

• Personalised medicine tailors prevention, diagnosis, monitoring of disease progression, treatment, and treatment response to each patient

• Identifying genetic variants enables early detection and targeted therapy

• Gene-based therapies and newborn screening transform disease outcomes

• Advances in cancer genetics and pharmacogenomics guide safer and more precise care

• Laboratory diagnostics translates genomics into real clinical benefits

## Introduction

Personalised medicine has become a central paradigm in modern healthcare, aiming to tailor prevention, diagnosis, and treatment to each patient’s unique molecular and clinical profile (*1*). Hereditary disorders clearly illustrate this progress: detecting disease-causing variants allows precise risk assessment, presymptomatic diagnosis, and targeted therapeutic strategies (*2*). In terms of outcome modifications, an excellent example is development of cystic fibrosis transmembrane conductance regulator (CFTR) modulators which shows how innovative therapies can fundamentally alter a disease’s course-turning cystic fibrosis from an inevitably fatal childhood disorder into a manageable chronic condition (*3*). Traditionally based on biochemical assays, newborn screening programs are now adopting genomic technologies. This shift enables presymptomatic diagnosis and treatment of conditions such as spinal muscular atrophy, where early intervention markedly improves survival and motor function (*4*, *5*). Similarly, advances in cancer genetics have revealed how germline mutations in cancer susceptibility genes are associated with cancer risk, enabling risk-appropriate screening and management recommendations, and how the synergy between germline and somatic mutations drives hereditary malignancies.

Precision oncology now applies molecular profiling to match patients with targeted therapies and innovative clinical trials (*6*, *7*). Pharmacogenomics further advances personalization by guiding therapy selection, predicting drug response and toxicity, and integrating complex drug-gene-environment interactions into routine clinical practice (*8*, *9*).

## Hereditary disorders – Yesterday, today, and tomorrow

Over the past few decades, rapid advances in gene analysis technologies have transformed research into genetic disorders-uncovering their molecular mechanisms and enabling precise diagnostic and prognostic testing. Our understanding of these conditions has deepened in parallel with advances in genetics and molecular biology. Key milestones in the development of genetic technologies are shown in Table 1. The study of hereditary disorders dates back to the 19th century, when Gregor Mendel, often regarded as the father of genetics, described the principles of inheritance through experiments on pea plants. However, a real understanding of the molecular basis of inheritance emerged in the mid-20th century. A key milestone was the discovery of deoxyribonucleic acid (DNA) structure in 1953 by James Watson and Francis Crick. This breakthrough was based on X-ray crystallography data previously obtained by Rosalind Franklin (*10*, *11*). Watson and Crick received the Nobel Prize in Physiology for this discovery. Uncovering the double-helix DNA structure made it possible to understand how genetic information is stored and transmitted. In the following decades, scientists identified numerous genes related to hereditary diseases, such as cystic fibrosis, Huntington’s disease, thalassemia, sickle cell disease, phenylketonuria, and spinal muscular atrophy. Traditional methods such as Sanger sequencing, while accurate, were slow and limited in throughput (*12*). At the beginning of the 21st century, a revolution in genetics came with next-generation sequencing (NGS), which allows millions of DNA fragments to be read simultaneously. This made it technically feasible and financially accessible to analyse parts of the genome or even whole genomes. As a result, diagnosing rare hereditary diseases, discovering new genes and mutations, and understanding the genetic basis of complex disorders became much more achievable. The speed, sensitivity, and decreasing costs of these methods have made them the foundation of modern genetic diagnostics and research (*13*). Today, hereditary disorders are recognized as an important public health issue. People with a genomic predisposition to certain diseases, such as hereditary breast cancer, can take preventive measures early. Prenatal diagnostics and preimplantation genetic testing also allow for the detection of hereditary diseases even before birth. Gene therapy has emerged as one of the most promising areas of development, aiming to correct or replace faulty genes. New technologies, for example, clustered regularly interspaced short palindromic repeats-associated protein 9 (CRISPR-Cas9) offer the potential for precise genome editing but also raise important ethical questions (*14*). For now, treatments using these technologies are not part of routine clinical practice and remain largely within the realm of scientific research. Genomic newborn screening (gNBS) is also on the horizon, allowing for the detection of genetic variants associated with serious but potentially treatable hereditary diseases before clinical symptoms appear. While it improves diagnostic sensitivity compared to standard techniques, its implementation requires careful consideration of legal, ethical, and social issues. This includes informed consent, protection of genetic data, and especially the clinical interpretation of variants of uncertain significance (*15*). Looking ahead, further advancements in personalised medicine are expected, with therapies tailored to patients’ individual genetic profiles. The digital analysis of large genetic datasets, combined with artificial intelligence, will help predict disease risk and guide the selection of the most effective therapies for newly diagnosed patients (*16*). Through real-world examples of diagnostics and treatment, this review highlights the benefits achieved through the remarkable progress in genetics over the past two decades.

**Table 1:** Key milestones in the development of genetic technologies

**Time Period**	**Technology / Discovery**	**Significance**
1860s	Mendel’s laws of inheritance	Foundation of modern genetics
1953	Discovery of DNA structure	Understanding storage and transmission of genetic information
1977	Sanger DNA sequencing	First accurate method for reading DNA sequences
1990 – 2003	Human Genome Project	Complete mapping of the human genome
2005 – present	NGS	Fast, cost-effective genome analysis
From 2012	CRISPR-Cas9 genome editing	Precise genome modification; potential therapeutic applications
2020s	gNBS, AI in genomics	Early diagnosis, personalised medicine, data-driven treatment decisions
AI - Artificial intelligence. CRISPR-Cas9 - clustered regularly interspaced short palindromic repeats-associated protein 9. DNA - deoxyribonucleic acid. gNBS - genomic newborn screening. NGS - next-generation sequencing.		

## Cystic fibrosis and CFTR modulators – New drugs, new disease?

Cystic fibrosis (CF) is an autosomal recessive genetic disease caused by pathogenic variants in the *CFTR* gene which encodes the CFTR chloride channel protein. This defect leads to abnormal mucus production and results in a multisystem disease, with progressive lung failure being the leading cause of mortality among CF patients. Cystic fibrosis transmembrane conductance regulator dysfunction produces thickened secretions that primarily affect the lungs but also involve the pancreas and other organs (*12*).

Until recently, CF therapy focused on symptomatic treatment without targeting the underlying protein defect. Symptomatic treatment, including airway clearance techniques, pancreatic enzyme replacement, and antibiotics, has significantly improved survival but could not fully prevent ongoing lung damage and disease progression (*13*).

Following the discovery of the *CFTR* gene and its pathogenic variants, years of research led to the development of targeted therapies known as CFTR modulators. Cystic fibrosis transmembrane conductance regulator modulators are innovative therapies that act directly on the defective CFTR protein to restore its function, addressing the root cause of the disease rather than its consequences. Key CFTR modulators include ivacaftor, the dual combinations lumacaftor-ivacaftor and tezacaftor-ivacaftor, and the triple combinations elexacaftor-tezacaftor-ivacaftor and vanzacaftor-tezacaftor-deutivacaftor (Table 2). The triple combination therapy has been a major breakthrough, showing efficacy in approximately 90% of CF patients with eligible *CFTR* mutations, especially those with the *CFTR F508del* variant (NM_000492.4:c.1521_1523delCTT; p.Phe508del). Clinical trials and real-world data have demonstrated significant improvements in lung function, a decrease in pulmonary exacerbations, better nutritional status, and enhanced quality of life. Decreased sweat chloride levels, an important biomarker, indicate restored CFTR function. Moreover, early introduction of therapy may alter the natural course of CF and improve long-term outcomes (*14*, *15*).

**Table 2:** Overview of cystic fibrosis transmembrane conductance regulator modulators: mechanism of action, target mutations and European medicines agency - approved age indications

**CFTR modulator**	**Type**	**Mechanism of action**	**Targeted mutations**	Indicated age for therapy (EU/EMA)*
Ivacaftor (Kalydeco)	Potentiator	Increases CFTR channel opening probability	Gating mutations (*e.g. G551D*)	> 4 months
Lumacaftor–Ivacaftor (Orkambi)	Corrector + potentiator^†^	Improves CFTR folding and channel gating	Homozygous *F508del*^‡^	> 2 years
Tezacaftor–Ivacaftor (Symkevi)	Corrector + potentiator	Improves CFTR processing and gating	Homozygous *F508del* or one responsive mutation	> 6 years
Elexacaftor– Tezacaftor–Ivacaftor (Kaftrio)	Triple therapy	Two correctors plus one potentiator; significantly improves CFTR function	≥ 1 *F508del* allele	> 2 years
Vanzacaftor– Tezacaftor– Deutivacaftor (*in clinical trials*)	Triple therapy (next-generation)	Enhanced potency and improved pharmacokinetics	≥ 1 *F508del* and other mutations (*pending approval*)	In clinical trials
Commercial brand names are provided in parentheses. *Indicated age refers to the minimum approved age for therapy according to the European medicines agency (EMA). ^†^Potentiators increase the open probability of the CFTR channel at the cell surface, whereas correctors improve CFTR protein folding, processing and trafficking to the plasma membrane. ^‡^*F508del* refers to the *CFTR* variant NM_000492.4:c.1521_1523delCTT (p.Phe508del), the most common pathogenic mutation in cystic fibrosis. CFTR - cystic fibrosis transmembrane conductance regulator.				

The remarkable improvements in clinical outcomes achieved with CFTR modulators have led some experts to define cystic fibrosis as a “new disease”. Although the prognosis of CF has improved significantly, new challenges have emerged, such as long-term drug safety, high treatment costs, and treatment adherence (*16*).

Croatia is among European countries with a high prevalence of *CFTR F508del* variant, present in approximately 70-80% of CF alleles, making the majority of Croatian patients eligible for CFTR modulator triple therapy. These modulators have been introduced into clinical practice in Croatia since autumn 2021. According to national data, patients on triple therapy have shown significant improvements in both clinical outcomes and quality of life (*17*-*19*). Current national priorities for CF therapy include expanding eligibility for the triple combination of elexacaftor, tezacaftor, and ivacaftor to younger age groups and introducing vanzacaftor/tezacaftor/deutivacaftor into Croatian clinical practice.

## Newborn screening in the era of personalised medicine

A novel approach in medicine is focused on the specific needs of each patient and is described by the term “P4 medicine” (predictive, preventive, personalised, participatory). This concept is based on understanding an individual’s genetic features, biomarkers, environmental, and lifestyle factors (*20*). This knowledge enables clinicians to select the most appropriate preventive or therapeutic approach at the optimal time. At the same time, an increasing number of novel therapeutic options are becoming available for rare diseases, including gene therapies that are already in routine use for certain diseases. However, these therapies are most effective when started early-ideally during the presymptomatic phase of the disease (*21*).

Newborn screening (NBS) from dried blood spots (DBS) is a diagnostic approach widely recognized as one of the greatest achievements of modern public health, particularly in rare diseases (*22*). The fundamental goal of NBS is the early identification of newborns with rare monogenic disorders for which treatment exists and where early intervention reduces morbidity and mortality. For decades, Wilson and Jungner’s principles guided decisions on which diseases to include in NBS programs (*23*). However, advances in technology and clinical practice have required modifications to these historic criteria (*24*, *25*). The number of disorders included in NBS programs differs between countries and may now reach up to 50, depending on local policies (*26*). In Croatia, NBS was first introduced in 1978 with screening for phenylketonuria, followed by congenital hypothyroidism seven years later. Since 2017, tandem mass spectrometry (MS/MS) has been used in the Croatian NBS program, expanding screening to eight rare diseases. In 2023, molecular technologies were introduced, enabling screening for spinal muscular atrophy (SMA). An overview of the development of NBS in Croatia is presented in Table 3.

**Table 3:** Milestones in the implementation of newborn screening in Croatia

**Year**	Test/Condition introduced*	**Technology used^†^**
1978	Phenylketonuria	Biochemical test from DBS
1985	Congenital hypothyroidism	Hormone analysis
2017	Eight rare diseases	Tandem mass spectrometry
2023	Spinal muscular atrophy	Molecular methods (PCR)
2023	Expanded Croatian NBS panel	Customized NGS panel (confirmatory testing)
*Refers to the condition(s) newly included in the national newborn screening (NBS) programme in the specified year. ^†^Technology primarily used for screening or confirmatory testing at the time of implementation. DBS - dried blood spots. NBS - newborn screening. NGS - next-generation sequencing. PCR - polymerase chain reaction.		

Tandem mass spectrometry enables rapid detection of numerous metabolites in DBS and has transformed NBS from single-disease testing into a platform capable of identifying more than 40 conditions simultaneously. Today, MS/MS is widely used as a first-tier test for inborn errors of metabolism (IEM). Nevertheless, it has well-known limitations, including sensitivity to sample quality, the risk of false-positive and false-negative results, and the inability to detect disorders without measurable metabolite changes in blood (*27*). The strengths and limitations of different NBS technologies are summarized in Table 4.

**Table 4:** Technologies in newborn screening – advantages, limitations and clinical applications

**Technology**	**Advantages**	Limitations*	**Application**
MS/MS	Rapid multi-metabolite analysis; detects > 40 conditions simultaneously	Sample-quality sensitive; risk of FP/FN results; limited to metabolite-detectable disorders	IEM, first-tier NBS
gNBS (PCR, MLPA, ddPCR)	Direct variant detection; fast and precise	Limited to predefined target genes; requires prior assay validation	SMA, SCID
NGS	Wide gene–disease coverage (~480 genes)	High cost; challenges in VUS interpretation; requires advanced bioinformatics expertise	Confirmatory testing after positive NBS results
Untargeted metabolomics	Discovery of novel biomarkers; deeper phenotypic insight	Lack of standardization; requires extensive clinical validation	Potential future application
*These represent potential limitations of screening technologies, reflecting the possibility of incorrect classification due to analytical constraints, biological variability, or incomplete genomic coverage. ddPCR - droplet digital polymerase chain reaction. FP - false positive. FN - false negative. gNBS - genomic newborn screening. IEM - inborn errors of metabolism. MLPA - multiplex ligation-dependent probe amplification. MS/MS - tandem mass spectrometry. NBS - newborn screening. NGS - next-generation sequencing. PCR - polymerase chain reaction. SCID - severe combined immunodeficiency. SMA - spinal muscular atrophy. VUS - variant of uncertain significance.			

The extraction of genetic material from DBS has enabled gNBS, now widely adopted in Europe as a first- or second-tier test for SMA and severe combined immunodeficiency. Methods used include real-time polymerase chain reaction (PCR), digital droplet PCR, and multiplex ligation-dependent probe amplification (*28*). In parallel, the development of NGS has accelerated progress in genetics. Following positive biochemical screening results, a customized NGS panel is now used as a confirmatory diagnostic test for all disorders included in the Croatian NBS program (*29*).

In the current era of genomic medicine, NBS faces new challenges. The gNBS could significantly expand the number of screened conditions if used as a first-tier test, while reducing false positives, shortening diagnostic times, improving predictive values, and broadening clinical utility (*30*). Several international pilot studies are evaluating the feasibility of gNBS panels, which typically include a median of 480 gene-disease associations (*31*). However, broader screening does not automatically mean a better NBS program. Integration of molecular technologies into NBS offers numerous advantages, but also raises questions such as legal and ethical concerns, interpretation of results, and infrastructural requirements, as summarized in Table 5. There is also a need for effective systems for long-term follow-up and care for persons diagnosed with a genetic condition through NBS (*32*).

**Table 5:** Challenges in integrating genomic newborn screening into clinical practice in Croatia

**Category**	**Key challenges**
Ethical and legal	Data privacy in Croatia; informed consent procedures; potential psychological impact on families; long-term storage and secondary use of genomic data
Interpretation	Need for additional training of healthcare professionals; standardization of sequencing workflows; quality control; interpretation of VUS; reporting of predisposition genes
Infrastructure	Investment in laboratory and bioinformatics capacity; establishment of multidisciplinary teams; development of long-term clinical follow-up systems
Societal	Public and patient engagement; acceptance of genomic technologies; cost considerations and equity of access
The listed challenges reflect current organizational, regulatory and infrastructural aspects relevant to the implementation of genomic newborn screening within the Croatian national healthcare system, based on available national programme data and implementation experience. VUS - variant of uncertain significance.	

At the same time, untargeted metabolomics using quadrupole time-of-flight (Q-TOF) or tandem Orbitrap instruments is emerging as a complementary approach. This approach offers greater insights into phenotypes of disease and can detect novel and known disease biomarkers. However, current methods still need comprehensive validation and careful selection of clinically significant biomarkers for examination (*33*).

Today and in the near future, designing an effective NBS demands strategic combination of advanced metabolomics with biochemical methods, and genomic technologies in standardized protocols. Such approaches also require the availability of appropriate therapeutic options for the screened diseases. Current consensus suggests that the introduction of genomic methods into NBS should be justified only for disorders with early childhood onset and for diseases with effective treatments that can cure, prevent, or slow disease progression (*34*, *35*). The future direction of NBS is closely linked to personalised medicine, integrating genetic findings into individualized treatment and follow-up strategies for each infant. It will require integration of NBS with health information systems, artificial intelligence systems, and longitudinal follow-up of infants identified through NBS to track their long-term health outcomes and treatment effectiveness. The multi-omics approach offers exciting potential to expand the scope of NBS programs in the future (*36*).

Along with this new potential, sustaining, and advancing NBS programs present an ongoing challenge. Despite all current and future technological possibilities, the benefits of NBS for the child must always outweigh the risks. Any revision of NBS criteria should include medical, legal, ethical, and social considerations. Furthermore, it should engage patients, key stakeholders, and the wider community.

## Spinal muscular atrophy in newborn screening - Treatment advantages

Spinal muscular atrophy shows how NBS programmes can improve disease outcomes through early treatment initiation (*29*, *37*). It is a severe neuromuscular disorder most often caused in about 95% of cases by homozygous deletion of the *SMN1* gene, and is characterized by high morbidity and mortality, particularly in its most severe form – Type 1 (*38*, *39*). The introduction of disease-modifying therapies has revolutionised the therapeutic landscape of SMA, shifting the focus from palliative care to a promising curative strategy when treatment begins before symptom onset (*40*, *41*). Implementing NBS for SMA enables early identification of affected infants before symptoms appear, providing a unique opportunity for early medical intervention (*29*, *37*). Presymptomatic therapy yields the best outcomes, as motor neurons remain responsive to therapy before irreversible damage occurs (*42*, *43*).

### Revolutionary therapeutic landscape

Three ground-breaking therapies have transformed outcomes for SMA patients: nusinersen, onasemnogene abeparvovec, and risdiplam (*40*, *42*, *43*). Each therapy targets the core pathophysiology of SMA. Nusinersen, administered intrathecally, is an antisense oligonucleotide that modulates *SMN2* pre-mRNA splicing to enhance the synthesis of functional SMN protein (*40*, *44*). Clinical studies show remarkable efficacy when initiated presymptomatically, with patients achieving motor milestones that sharply contrast with the natural history (*40*, *42*). The NURTURE study reported 100% survival without permanent ventilation and near-normal motor development in most participants treated presymptomatically (*40*, *45*). Onasemnogene abeparvovec, the first gene therapy for SMA, delivers a functional *SMN1* gene copy *via* an adeno-associated virus vector; when administered presymptomatically, infants reach milestones typically unattainable in untreated SMA Type 1 (*43*). Risdiplam is an orally administered small molecule that enhances *SMN2* pre-mRNA splicing and increases production of functional SMN protein. Its oral bioavailability and favourable safety profile support long-term therapy, with best results when started presymptomatically (*40*, *43*). The three available disease-modifying therapies are summarised in Table 6.

**Table 6:** Disease-modifying therapies for spinal muscular atrophy

**Therapy**	**Type**	Drug administration*	**Mechanism** **of action**	**Key** **evidence^†^**
Nusinersen	Antisense oligonucleotide	Intrathecal injection	Modification of *SMN2* pre-mRNA splicing leading to increased production of functional SMN protein	NURTURE study: presymptomatically treated infants showed survival and attainment of motor milestones not typically observed in untreated SMA type 1
Onasemnogene abeparvovec	Gene therapy	Intravenous infusion (single dose)^‡^	AAV9-mediated delivery of a functional *SMN1* gene resulting in increased SMN protein expression	Presymptomatic infants achieved major motor milestones rarely observed in untreated SMA type 1
Risdiplam	Small molecule	Oral administration	Modification of *SMN2* splicing resulting in increased systemic SMN protein levels	Demonstrated sustained motor function improvement, with greatest benefit when initiated presymptomatically
*Drug administration indicates the route and mode of delivery used in clinical practice. ^†^Key evidence summarizes findings from pivotal clinical trials supporting the efficacy of early or presymptomatic treatment. ^‡^ “Single dose” refers to one-time systemic gene replacement therapy administered as a single intravenous infusion. AAV9 - adeno-associated virus vector 9. SMA - Spinal muscular atrophy. SMA type 1 - most severe early-onset form of SMA characterized by symptom onset in infancy and rapid motor neuron degeneration. SMN - Survival motor neuron.				

The advantage of presymptomatic over symptomatic treatment is substantial and consistent across all available therapies (*40*, *42*). Natural history studies show that patients with SMA Type 1 rarely achieve independent sitting, with a median survival without ventilatory support of ~10.5 months (*38*). In contrast, presymptomatically treated patients frequently achieve independent sitting, standing, and even walking (*40*, *42*, *43*). Long-term follow-up confirms durable benefits: treated patients show normal growth, preserved swallowing, fewer respiratory complications, and markedly improved quality of life (*43*, *46*, *47*). While symptomatic patients may respond, they seldom reach the developmental milestones seen with presymptomatic treatment (*40*, *42*). The therapeutic window is therefore critical; delays in therapy initiation can compromise outcomes (*41*-*43*). A comparison of natural history *versus* presymptomatic outcomes is shown in Table 7.

**Table 7:** Clinical outcomes in spinal muscular atrophy: natural history *vs.* presymptomatic treatment

**Outcome**	**Natural history** **(SMA Type 1)**	**Presymptomatic** treatment*
Survival	Median survival 10.5 months without permanent ventilatory support	> 95% survival without need for permanent ventilation
Motor milestones	Independent sitting rarely achieved	Independent sitting, standing and walking frequently achieved when treatment is initiated presymptomatically
Respiratory function	Recurrent infections and early respiratory failure	Preserved respiratory function with fewer complications
Quality of life	Severe motor disability and high caregiver burden	Improved growth and swallowing, reduced disease burden and better family well-being
This comparison summarizes published evidence on the natural history of untreated SMA type 1 and outcomes observed in infants receiving disease-modifying therapy initiated during the presymptomatic stage (46,47). Outcomes are based on clinical trial and observational cohort data demonstrating substantially improved survival and motor development with early treatment. SMA - spinal muscular atrophy.		

The Croatian SMA NBS pilot study, conducted at the University Hospital Centre Zagreb, established a clinical pathway from screening-to-treatment (*29*). During one year of the study, five SMA patients were identified among 32,655 screened newborns. All five patients immediately received specialised neuromuscular evaluation and rapid therapy introduction. The success of the pilot study is reflected both in analytical performance and in the clinical outcomes achieved through early therapy initiation. All infants identified in the first year began treatment before symptom onset, with constant monitoring demonstrating preserved motor function and age-appropriate milestones (*29*). This real-world experience verifies the benefits of presymptomatic treatment observed in clinical trials (*40*, *42*, *43*). The multidisciplinary approach ensures a seamless transition from screening to treatment and serves as an outline for effective clinical implementation (*29*). The Croatian pilot results are summarised in Table 8.

**Table 8:** Croatian spinal muscular atrophy newborn screening pilot study (2022–2023)

**Parameter**	**Results**
Period	1 year
Number of newborns screened	32,655
SMA patients identified	5
Time of diagnosis	Before symptom onset
Intervention	Immediate specialist evaluation plus early therapy
Outcome	Preserved motor function; age-appropriate milestones
This table summarizes the initial real-world outcomes of implementing newborn screening for spinal muscular atrophy, demonstrating early identification of affected infants and timely initiation of therapy prior to symptom onset, which is associated with improved motor development and clinical prognosis. The data reflect results from the national newborn screening programme in Croatia during the first year of SMA screening implementation (29). SMA - spinal muscular atrophy.	

Furthermore, newborn screening supports a personalised-medicine approach that integrates genetic and clinical factors. Examination of *SMN2* gene copy number leads treatment intensity, as fewer copies generally predict more severe disease type (*39*, *44*). Newly developed assessment tools, including motor scales for presymptomatic infants and biomarkers such as compound muscle action potentials, enable accurate monitoring of treatment response and disease progression (*40*, *42*).

### Economic and societal impact

Patients treated presymptomatically require fewer hospitalisations, fewer respiratory interventions, and less supportive care than those treated after symptom onset (*40*, *46*). Preservation of motor function mitigates long-term disability-related costs and extends productive years of life. Family quality of life improves substantially with presymptomatic therapy, reducing caregiver burden and enhancing family functioning (*46*).

Addition of SMA to the existing national screening programme in Croatia, together with modern SMA treatments, demonstrates precision medicine in action: early recognition leads to transformative clinical outcomes (*40*, *41*, *43*). The results of the Croatian pilot study confirm that well-organised screening-to-treatment pathways deliver the benefits of presymptomatic intervention in real-world clinical practice (*29*). The body of evidence supports NBS for SMA as a vital component of comprehensive care, ensuring affected infants receive life-changing therapies at the optimal time (*40*, *42*, *43*).

## Hereditary malignant diseases

Hereditary malignant diseases often present as hereditary cancer syndromes (HCSs), groups of cancers caused by inherited, germline pathogenic variants that typically follow recognizable clinical patterns and affect multiple organs. Rare congenital disorders, such as Bloom syndrome, Fanconi anemia, and ataxia-telangiectasia, show broader multi-organ involvement and are usually inherited in an autosomal-recessive pattern, caused by biallelic mutations in DNA-repair genes. These often present with immunodeficiency and early-onset cancers (*48*, *49*). In contrast, most individuals with HCSs appear clinically healthy, except for an increased lifetime cancer risk due to inherited germline pathogenic variants (GPVs) in key genes such as tumour suppressors, oncogenes, or mismatch repair (MMR) genes. These GPVs typically follow autosomal dominant inheritance, with a 50% chance of transmission. Although a single inherited GPV does not directly cause cancer because cellular repair mechanisms remain intact, only a few additional somatic mutations are needed to trigger malignant transformation, leading to earlier cancer onset than in the general population.

Hereditary cancer syndromes account for about 10% of all malignancies, yet many remain undiagnosed. Common indicators include early-onset cancers, multiple primary tumours, family history, and rare presentations like male breast cancer (*50*-*52*).

Most HCS-related genes are tumour suppressors (*e.g*., *BRCA1 –* breast cancer 1; *BRCA2* – breast cancer 2; *MLH1 –* mutL homolog 1*; MSH2 –* mutS homolog 2; *RB1 –* RB transcriptional corepressor 1), where cancer typically develops after a “second hit” inactivates the remaining functional allele. A smaller number of HCSs involve inherited activating mutations in oncogenes, such as *RET* (ret proto-oncogene receptor tyrosine kinase) in multiple endocrine neoplasia types 2A and 2B (*53*). A single gene can predispose to several cancer types, while the same cancer may arise from mutations in different genes (Table 9). For example, GPVs in *MMR* genes cause Lynch syndrome (colorectal, endometrial, ovarian, gastric cancers), while *TP53* (tumour protein p53) and *CHEK2* (checkpoint kinase 2) genes are linked to Li-Fraumeni syndrome (leukaemia, breast cancer, sarcoma, brain tumours). Pathogenic variants in the *BRCA1* and *BRCA2* gene are responsible for hereditary breast and ovarian cancer syndrome, increasing the risk of breast, ovarian, prostate, and pancreatic cancers. Beyond *BRCA1* and *BRCA2*, variants in genes like *ATM* (ataxia-telangiectasia mutated), *PALB2* (partner and localizer of BRCA2), *BRIP1* (BRCA1 interacting helicase 1), *STK11* (serine/threonine kinase 11), and *NBN* (nibrin) also contribute to a moderate hereditary predisposition for breast cancer (*51*, *52*).

**Table 9:** Hereditary cancer predisposition syndromes in clinical oncology

**Syndrome**	**Tumour type**	**Mode of inheritance**	**Genes**
Hereditary breast cancer and ovarian cancer syndrome	Breast cancer Ovarian cancer Prostate cancer Pancreatic cancer	Dominant	*BRCA1* *BRCA2*
	Fanconi anemia/medulloblastoma	Recessive	*BRCA2*
HNPCC, including ‘‘Lynch II’’ syndrome	Colon cancer Endometrial cancer Ovarian cancer Renal pelvis cancers Ureteral cancers Pancreatic cancer Stomach and small bowel cancers Hepatobiliary cancers	Dominant	*MLH1* *MSH2* *MSH6*
Li-Fraumeni Syndrome	Soft tissue sarcoma Breast cancer Osteosarcoma Leukaemia, Brain tumours Adrenocortical carcinoma	Dominant	*TP53* *CHEK2*
Cowden Syndrome	Breast cancer Thyroid cancer Endometrial and other cancers	Dominant	*PTEN*
FAP, AFAP	Colon cancer	Dominant	*APC*
MAP	Colon cancer	Recessive	*MUTYH*
Hereditary gastric cancer	Stomach cancers	Dominant	*CDH1*
Juvenile polyposis	Gastrointestinal cancers Pancreatic cancer	Dominant	*SMAD4* *BMPR1A*
Peutz-Jeghers syndrome	Colon cancer Small bowel cancer Breast cancer Ovarian cancer Pancreatic cancer	Dominant	*STK11*
Familial gastrointestinal stromal tumour	Gastrointestinal stromal tumours	Dominant	*KIT*
Hereditary melanoma pancreatic cancer syndrome	Pancreatic cancer Melanoma	Dominant	*CDKN2A*
Bloom syndrome	Leukaemia Carcinoma of the tongue Squamous cancers Wilms’ tumour Colon cancer	Recessive	*BLM*
Nijmegen breakage syndrome	Lymphoma Glioma Medulloblastoma Rhabdomyosarcoma	Recessive	*NBS1*
Retinoblastoma	Retinoblastoma Osteosarcoma	Dominant	*RB1*
Hereditary paraganglioma	Paraganglioma Pheochromocytoma	Dominant	*SDHD* *SDHC* *SDHB*

This table provides a selective overview of clinically relevant hereditary cancer predisposition syndromes in oncology and is not intended to represent an exhaustive list of all known syndromes.

AFAP - attenuated familial adenomatous polyposis. *APC* - adenomatous polyposis coli. *BLM* - Bloom syndrome RecQ like helicase. *BMPR1A* - bone morphogenetic protein receptor type 1A. *BRCA1* - breast cancer 1. *BRCA2* - breast cancer 2. *CDH1* - cadherin 1. *CDKN2A* - cyclin-dependent kinase inhibitor 2A. *CHEK2* - checkpoint kinase 2. FAP - familial adenomatous polyposis. HNPCC - hereditary nonpolyposis colorectal cancer. *KIT* - KIT proto-oncogene receptor tyrosine kinase. MAP - MUTYH-associated polyposis. *MLH1* - mutL homolog 1. *MSH2* - mutS homolog 2. *MSH6* - mutS homolog 6. *MUTYH* - mutY DNA glycosylase. *NBN* (NBS1) - nibrin. *PTEN* - phosphatase and tensin homolog. *RB1* - RB transcriptional corepressor 1. *SDHB* - succinate dehydrogenase complex iron sulfur subunit B. SDHC - succinate dehydrogenase complex subunit C. *SDHD* - succinate dehydrogenase complex subunit D. *SMAD4* - SMAD family member 4. *STK11* - serine/threonine kinase 11. *TP53* - tumour protein p53.

It is important to emphasize that individuals do not inherit cancer itself but rather GPVs in specific genes that increase susceptibility to certain tumour types. These variants are classified according to their penetrance into high, moderate and low-risk categories. High-risk (highly penetrant) genes, such as *BRCA1*, *BRCA2*, *MLH1*, *MSH2*, *MSH6*, *APC* and *TP53*, are linked with HCSs such as HBOC, Lynch syndromes, FAP and Li-Fraumeni syndrome. Individuals with GPVs in these genes may have a 5 to 50-fold increased risk compared to the general population. Moderate-risk genes, including *ATM*, *BRIP1*, *CHEK2* and *PALB2*, give a 1.5 to 5-fold increased cancer risk. Low-risk variants, while individually associated with only a slight increase in risk (up to 1.5-fold), are common in the population, and their cumulative effect may be clinically relevant (*54*).

The molecular basis of hereditary malignant diseases lies in GPVs within the genome that affect gene function or expression. The American college of medical genetics (ACMG) has established a five-tier system classifying genetic variants: pathogenic (P), likely pathogenic (LP), of uncertain significance (VUS), likely benign (LB), and benign (B) (*55*). In oncology, the International Agency for research on cancer (IARC) further refines this by applying quantitative probability thresholds to assess disease causality (Table 10) (*56*). These classification schemes lead the clinical interpretations of genetic results. Gene variants graded as P or LP are counted as clinically actionable, they give information about treatment and disease-specific surveillance. However, VUSs are still a key challenge due to their doubtful impact on gene function and risk of disease. Continuous follow-up and genetic counselling are therefore essential for such patients so determination of VUS clinical relevance over time can be done. The cancer development has a multifactorial basis, individuals with suspected HCSs are recommended to take genetic testing (NGS and multi-gene panels focused on cancer susceptibility genes). Compared to traditional methods, NGS is faster, broader, and more cost-effective. It can be used for single-genes, targeted panels, whole exomes, or even whole genomes, giving complete genetic information but keeping controllable costs (*57*, *58*). In oncology, multi-gene panel NGS testing is favoured due to its higher analytical sensitivity and specificity. By focusing on disease-relevant genes, it simplifies interpretation and improves diagnostic accuracy. Genetic testing identifies GPVs that increase cancer risk and has several key clinical applications (*59*). First, certain variants predict treatment response, for example, *BRCA1* and *BRCA2* gene variants indicate potential benefit from PARP inhibitors, while *MMR* gene variants can guide immunotherapy decisions (*60*, *61*). Second, confirming a HCS at the molecular level enables tailored preventive strategies, including enhanced surveillance or prophylactic surgery, which can significantly improve outcomes (*62*, *63*). Third, identifying a GPV in a patient allows for cascade testing in family members, facilitating individualized preventive or therapeutic plans (*64*, *65*).

**Table 10:** Classification system of genetic variants according to the International agency for research on cancer

Class*	**Description** ^†^	**Probability of** **being pathogenic**	**Counseling consequences** ^‡^
1	Benign	< 0.001	Considered equivalent to “no pathogenic variant detected”; routine population-based screening recommended
2	Likely benign	0.010-0.049	Managed as negative test result; standard clinical surveillance based on general risk
3	VUS	0.050-0.949	Clinical management guided by personal and family history; periodic variant re-evaluation
4	Likely pathogenic	0.950-0.990	Genetic counselling and intensified surveillance according to high-risk management guidelines
5	Pathogenic	> 0.990	Full high-risk management including tailored surveillance, preventive strategies and cascade testing of relatives
*Classes refer to the IARC five-tier classification of germline genetic variants in cancer susceptibility genes (56). ^†^Description indicates the clinical interpretation of the variant classification. ^‡^Counselling consequences refer to recommendations for genetic counselling, clinical surveillance and risk management of the proband and at-risk family members. IARC - International agency for research on cancer. VUS - variant of uncertain significance.			

All genetic testing candidates must take pre-test counselling and give informed consent. Counselling should cover the purpose and limitations of the genetic test, likely outcomes, implications for family members and privacy concerns.

The discovery of cancer-predisposing genes and improved diagnosis of hereditary malignancies represent significant progress in translational medicine today, in the current era of personalised medicine. Future investigations should spotlight strategies for tumour development prevention and progression prevention in germline pathogenic variants carriers.

## From genetics to treatment: a personalised approach to oncology

Precision oncology moves beyond the one-size-fits-all model of cancer care. It emphasises therapies customized to the patient’s genetic and molecular features and directed toward cancer-specific mutations (*66*, *67*). This approach is driven by the recognition that the same mutations can occur in tumours from different organs, allowing treatment efficacy to be independent of tumour origin. The goal is to improve outcomes by using treatments tailored to each patient’s unique genetic changes.

Cancer is a disease of cellular evolution. Over time, cancer cells acquire mutations that confer a survival advantage, enabling uncontrolled growth and resistance to cell death. This reflects a loss of control over the cell cycle, which is regulated by two major gene classes: proto-oncogenes and tumour-suppressor genes. Proto-oncogenes promote normal cell growth, in the case of the mutation, they become oncogenes, with a “gain of function” which leads to uncontrolled cell proliferation. A single mutated allele can be enough to alter gene function. These mutations are typically acquired. Tumour suppressor genes suppress cell growth and programmed cell death. Mutations in these types of genes result in a “loss of function” and commonly require both mutated alleles to be deactivated, often through loss of heterozygosity (LOH). A subclass, DNA repair genes, are tumour suppressors since their loss of function increases the overall mutation rate in other genes. The variations between proto-oncogenes, tumour suppressor, and DNA repair genes are summarised in Table 11.

**Table 11:** Gene categories in cancer

**Gene type**	**Normal role**	**Effect of mutation**	**Example genes**	**Inheritance pattern**
Proto-oncogenes	Promote growth/division	Gain of function → oncogene activation, and uncontrolled proliferation	*KRAS, BRAF,* *EGFR*	Somatic (predominantly)
Tumour suppressor genes	Inhibit growth, trigger apoptosis	Loss of function → uncontrolled cell growth	*TP53, RB1,* *APC*	Germline with somatic “second hit” or somatic only
DNA repair genes	Maintain genomic stability and DNA integrity	Loss of function → accumulation of mutations	*BRCA1, BRCA2, MSH2, MLH1*	Germline or somatic
*APC* - adenomatous polyposis coli. *BRAF* - B-Raf proto-oncogene, serine/threonine kinase. *BRCA1* - breast cancer 1. *BRCA2* - breast cancer 2. *EGFR* - epidermal growth factor receptor. *KRAS* - Kirsten rat sarcoma viral oncogene homolog. *MLH1* - mutL homolog 1. *MSH2* - mutS homolog 2. *RB1* - RB transcriptional corepressor 1. *TP53* - tumour protein p53.				

Key mutations, critical for a tumour’s growth and progression, are referred to as driver mutations. They are often point mutations and are rarely lost due to weak negative selection (*68*). Tumours typically have about four driver mutations, though this varies by cancer type (*69*). Driver mutations are characterized by their recurrence across many patients and at specific “hotspots” (*70*). Most other mutations are passenger mutations, which are not critical for tumour growth, but recent research suggests they can have a cumulative effect on the tumour’s characteristics (*71*).

Most tumour mutations are somatic (acquired) due to environmental factors or aging; only 5-10% are germline (inherited) (*72*). Unlike germline mutations, which are in every cell, somatic mutations are only in the cells where they occurred, leading to clonal heterogeneity. As a tumour grows, different subpopulations of cells, or subclones, develop unique mutations (*73*).

Cancer treatments act as a powerful selective force (*74*). A drug may kill the dominant, “naive” cancer cells, but it selects for and promotes the growth of a small fraction of cells that have acquired resistance mutations. This resistant subclone then becomes the dominant population, explaining why subsequent therapies are often less effective.

Mutations can be detected using tissue biopsy or a non-invasive liquid biopsy, which identifies tumour-derived DNA fragments circulating in the bloodstream (*75*). Liquid biopsies can detect mutations from multiple subclones.

Today, NGS is the primary method for analysing cancer genomics (*76*). It can detect various mutations, for example, single nucleotide polymorphisms (SNPs), small deletions and insertions (indels), and larger changes like copy number variations (CNVs). The variant allele frequency (VAF) helps differentiate between germline and somatic mutations; a high VAF (50-100%) suggests a germline mutation, while somatic mutations can have a very low VAF (*77*).

Somatic mutations leave characteristic patterns called mutational signatures, with more than 80 identified by COSMIC (*78*). These signatures fall into three types:

Type 1: caused by exogenous or endogenous processes (*e.g*., UV radiation).
Type 2: linked to defective DNA repair mechanisms (*e.g*., *BRCA1/BRCA2* mutations).
Type 3: caused by unknown processes.

The classification of mutational signatures and their therapeutic implications is presented in Table 12.

**Table 12:** Mutational signatures and their therapy implications

**Signature type**	**Main cause**	**Example**	**Therapy relevance**
Type 1	Exogenous and endogenous mutational processes	UV-induced mutations	May predict response to immunotherapy in melanoma
Type 2	DNA repair defects	*BRCA1/2* deficiency	Increased sensitivity to platinum-based chemotherapy and PARP inhibitors
Type 3	Unknown mechanisms	–	Currently under research
Signature types represent simplified categories of mutational processes associated with specific biological mechanisms and therapeutic vulnerabilities. *BRCA1* - breast cancer 1. *BRCA2* - breast cancer 2. PARP - poly(ADP-ribose) polymerase. UV - ultraviolet.			

Understanding these signatures is clinically valuable as a predictor of therapeutic response. For example, tumours with a specific signature indicating a defect in homologous recombination are sensitive to platinum-based therapies or PARP inhibitors. A high tumour mutational burden (TMB) signature or a defect in the mismatch repair (MMR) system that leads to high microsatellite instability (MSI-H) are both excellent predictors of response to immunotherapy, regardless of the tumour’s location (*79*).

Different to conventional chemotherapy, precision oncology drugs have high specificity for molecular targets (*80*). These drugs fall into two main categories: small-molecule drugs and biologics. Chemically synthesized small-molecule drugs act on intracellular or extracellular targets (*e.g*., kinase inhibitors), while biologic drugs, typically antibodies, target extracellular targets (*e.g*., cell receptor inhibitors). Although precision oncology has potential, it faces challenges such as tumour heterogeneity, considerable healthcare costs, and limited access to genetic testing (*81*). Consequently, to address these concerns, innovative clinical trial designs have been developed to better evaluate targeted therapies and enable their implementation in precision oncology (*82*, *83*). Table 13 summarizes contemporary adaptive clinical trial designs used in precision oncology to match targeted therapies with specific genomic alterations either across or within tumour types.

**Table 13:** Innovative clinical trial designs in precision oncology

**Trial design**	**Inclusion criteria**	**Purpose**	**Example**
N-of-1*	Single patient with a defined molecular alteration	Evaluation of individualized targeted therapy in a single patient	Rare tumours with unique actionable mutations
Basket	Different tumour types sharing the same molecular alteration	Assessment of efficacy of mutation-targeted therapy across cancer types	NTRK inhibitor trials
Umbrella	Single tumour type with different molecular subgroups	Allocation of targeted therapies based on molecular profiling	Lung cancer umbrella trials
Super-umbrella	Hybrid design combining basket and umbrella approaches	Large adaptive master protocol evaluating multiple targets and therapies	Master protocols in precision oncology
This table summarizes contemporary adaptive clinical trial designs used in precision oncology to match targeted therapies with specific genomic alterations across or within tumour types. *N-of-1 trial refers to a single-patient clinical study in which treatment is selected based on the individual molecular profile, allowing direct assessment of therapeutic benefit in that specific patient. NTRK - Neurotrophic tyrosine receptor kinase.			

All multifaceted decisions are often reached by a multidisciplinary team of experts.

Results of molecular profiling precisely direct the selection of targeted drugs that work on specific molecular pathways. Key patterns include:

Kinase and receptor mutations: tumours with *BRAF*, *EGFR*, and *ALK* gene mutations exhibit constant growth signals and can be treated with kinase inhibitors. Amplification of the *ERBB2* (*HER2*) gene is targeted with monoclonal antibodies (trastuzumab).
DNA repair mutations: tumours with *BRCA1*/*BRCA2* mutations have damaged DNA repair, and consequently are sensitive to PARP inhibitors. Tumours with a defect in the *MMR* system respond well to immunotherapy.
Other targets: mutations in genes such as *IDH1*, *PIK3CA*, *KRAS G12C*, *NTRK*, and *RET* can be treated with specific inhibitors.

Detailed molecular profiling, from driver mutations and mutational signatures to understanding tumour heterogeneity, is the foundation of personalised cancer treatment. As our knowledge of tumour genetics progresses, new targeted treatments continue to emerge. These advances have turned precision oncology into standard practice, allowing clinicians to tailor therapy decisions according to the patient’s unique genetic characteristics.

## Pharmacogenetics and individualisation of therapy

The concept of individualising drug therapy based on genetic information is one of the key implementations of personalised medicine today. Pharmacogenetics, the study of how genetic variation affects individual responses to therapy, aims to optimise drugs selection and minimise adverse drug reactions (ADRs). As early as the mid-20th century, discoveries of inherited enzyme insufficiencies, for example, glucose-6-phosphate dehydrogenase (G6PD) deficiency and N-acetyltransferase polymorphisms revealed that genetics significantly modulates drug metabolism and toxicity (*84*, *85*). Today, with the advent of NGS and bioinformatics, pharmacogenetics has expanded from single gene-drug interactions to pharmacogenomics, genome-wide approaches that consider the complex interplay of multiple genes and pathways (*86*, *87*). Currently, pharmacogenomics is clinically relevant for a growing number of medications, with actionable pharmacogenes guiding drug selection and dosing to improve efficacy and reduce ADRs. Implementation is supported by expert societies such as the Dutch pharmacogenetics working group (DPWG) and the clinical pharmacogenetics implementation consortium (CPIC), which have published evidence-based guidelines for more than 160 gene-drug pairs (*88*-*91*). Integrating pharmacogenetic data into clinical workflows has been associated with improved safety, efficacy, and greater cost-effectiveness, particularly for medicines with narrow therapeutic index or high ADR risk (*92*).

At the core of pharmacogenomics are inherited variants, especially in genes encoding drug-metabolising enzymes, transporters, receptors, drug targets, and immune modulators, that influence how individuals process and respond to medications throughout their lives (*86*, *87*, *93*). Therefore, pharmacogenomic testing focuses on germline sequence variants that influence individual variability in drug response, covering pharmacokinetics (drug metabolism) and pharmacodynamics (drug targets and sensitivity) (*94*). It is estimated that variations in genes involved in pharmacokinetics and pharmacodynamics explain about 20-30% of variability in drug response (*92*). Unlike inherited disease genetics, in pharmacogenetics germline sequence variants are not classified by the traditional pathogenicity categories used in medical genetics, because they primarily describe the impact on drug response and not disease causation. Major pharmacogenetics resources, such as PharmGKB (Pharmacogenomics knowledgebase), and newly established ClinPGx (Clinical pharmacogenomics), as well as CPIC and DPWG, classify gene-drug interactions by systematically evaluating drug-gene pair clinical relevance and actionability for drug prescribing decisions (*88*, *95*, *96*). These resources provide evidence-based recommendations regarding drug selection, dosage adjustment, or consideration of alternative therapies based on genotype-predicted phenotypes such as poor, intermediate, normal, or ultra-rapid metabolizer status, as well as enzyme activity scores and drug transporter functionality.

Genetic polymorphisms of cytochrome P450 (CYP) enzymes significantly influence individual responses to drugs, susceptibility to toxicity, and cancer risk. They are crucial for metabolising endogenous substances, xenobiotics, and carcinogens. Variants in CYP genes may lead to loss of enzyme function, reduced expression, altered substrate specificity, or increased enzyme activity (*97*, *98*). A key step in pharmacogenomics is translating genotype into phenotype, enabling functional predictions that guide drug therapy. For drug-metabolising enzymes such as CYPs, allele combinations define metabolic phenotypes of individuals, ranging from three to five categories depending on the specific enzyme and functional allele composition. These phenotypes include poor metabolizers (PMs; two nonfunctional alleles); intermediate metabolizers (IMs; one normal function and one nonfunctional allele, or two decreased function alleles), normal metabolizers (NMs; two normal function alleles, or one normal function and one decreased function allele), rapid metabolizers (RMs; one normal function and one increased function allele), and ultrarapid metabolizers (UMs; multiple active gene copies or two increased function alleles) (*94*, *99*). A similar genotype-to-phenotype translation applies to drug transporters (ABCG2, SLCO1B1), where variants alter substrate transport rather than metabolism. Functional categories range from poor, decreased, normal, to increased function phenotypes, influencing drug exposure and toxicity risk (*99*, *100*).

The most relevant pharmacogenomic markers are those with high-level evidence for clinical actionability by CPIC and DPWG, and those recognised by regulatory agencies such as the European medicines agency (EMA) and the United States Food and drug administration (FDA). The key pharmacogenomic genes include *ABCG2*, *CYP2B6*, *CYP2C9*, *CYP2C19*, *CYP2D6*, *CYP3A5*, *DPYD*, *HLA-B*, *NUDT15*, *SLCO1B1*, *TPMT*, *UGT1A1*, *VKORC1* and others (*85*, *86*, *88*, *89*, *91*, *95*). These genes are involved in the metabolism of numerous drugs, with common clinical applications across multiple therapeutic areas: psychiatry (selective serotonin reuptake inhibitors (SSRIs): *CYP2B6*, *CYP2C19*, *CYP2D6*); cardiology (clopidogrel: *CYP2C19*; mavacamten: *CYP2C19*; statins: *ABCG2*, *SLCO1B1*; warfarin: *CYP2C9*, *VKORC1*); oncology (fluoropyrimidines: *DPYD*; tamoxifen: *CYP2D6*; irinotecan: *UGT1A1*); pain management (opioids: CYP2D6); infectious diseases (HLA-B57:01 predicts abacavir hypersensitivity); immunosuppressive therapy (tacrolimus: *CYP3A5*; azathioprine: *NUDT15*, *TPMT*); and neurology (carbamazepine: *HLA-B15:02*; siponimod: *CYP2C9*), among others. Given the wide range of gene-drug interactions, transitioning from single-gene tests to pre-emptive pharmacogenomic panels is a logical next step. Recent large trials support this approach. The PREPARE study demonstrated that implementing a 12-gene pharmacogenetic panel significantly reduces ADRs across diverse European healthcare systems (*89*, *91*). This aligns with recent finding that 98.8% of individuals carry at least one actionable pharmacogenetic variant with therapeutic implications, and 23.3% have at least one specific gene-drug pair for which a therapy adjustment is recommended (*101*). This is particularly important for older adults and patients with polypharmacy, where pharmacogenetic testing can help to safely manage complex medication regimens and minimise ADRs in these vulnerable populations (*102*).

Pharmacogenetic information is increasingly incorporated into drug labels and clinical decision support tools, with more than 300 drug-gene pairs recognised by regulatory agencies FDA and EMA (*103*). Discrepancies exist between agencies in the level and specificity of pharmacogenetic recommendations, but harmonisation efforts are ongoing (*92*, *94*). The consensus of actionable pharmacogenomic labelling between the FDA and the EMA is about 50%, and guidelines provided by CPIC and DPWG are only partly implemented into the summary of product characteristics (SmPCs) (*104*). Pharmacogenomic actionable labelling is the most common classification, and *CYP2D6* most frequent gene in FDA pharmacogenomic labelling (*105*).

Both the efficacy and the safety of drug therapy vary among populations due to ethnogeographic differences in pharmacogenetic variants. These differences have direct implications for public health, supporting population-specific genotyping strategies and cost-effectiveness models for pharmacogenomic testing. However, there are significant inequities in global pharmacogenomic research coverage, with African and some Asian populations being underrepresented, despite their high genetic diversity and unique variant profiles (*92*, *106*). Recognising and integrating these differences is crucial for pharmacogenomic implementation in diverse patient groups (*86*).

While challenges remain in the implementation of pharmacogenomics into health-care systems – a knowledge gap in the health-care workforce, variability in reimbursement, integration with clinical decision systems, interpretation of rare variants - progress is steadily advancing (*90*, *92*). Beyond clinical use, pharmacogenomics also informs drug discovery and development (*86*). Expanding beyond single-gene testing to comprehensive, population-tailored strategies, supported by clinical guidelines, regulatory frameworks, and international collaboration, is essential for personalised medicine advancement.

## Antidepressant treatment in breast cancer patients using tamoxifen

Depression is a common and serious concern among breast cancer patients, affecting roughly one-third of women worldwide (*107*). Not only can depression arise from the psychological burden of having a life-altering and life-threatening illness, but the biological effects of cancer and its treatment can also cause depression. Among women in Croatia, breast cancer is the most frequently diagnosed cancer according to the Croatian Institute for Public Health. Almost 80% of patients with breast cancer have hormone receptor-positive disease, and in these patients, anti-oestrogen therapies are indicated as adjuvant treatment (*108*).

Tamoxifen, a weak anti-oestrogen prodrug, is metabolized into active metabolites, including 4-hydroxy-tamoxifen, N-desmethyltamoxifen, and 4-hydroxy-N-desmethyl-tamoxifen (endoxifen). The response to tamoxifen may vary among patients and depends partly on the *CYP2D6* genotype (*109*, *110*). The *CYP2D6* gene is highly polymorphic and is classified into four metabolic phenotypes: PM, IM, NM, and UM (*111*, *112*). The consequences of impaired tamoxifen metabolism are particularly significant in patients carrying decreased-function alleles (*CYP2D6* *9, *10, *41) or no-function alleles (*3, *4, *5, *6). The distribution of these variants differs across populations (*113*, *114*). Patients categorised as PM with inactivating alleles tend to have inferior survival *versus* carriers of the wild-type allele (*115*). Nevertheless, recent studies show inconsistent results regarding associations between *CYP2D6* alleles and clinical outcomes (*116*, *117*).

Individuals with low CYP2D6 enzyme activity, either due to genetic variants or concomitant usage of strong CYP2D6 inhibitors, show significantly decreased endoxifen concentrations during tamoxifen therapy (*118*, *119*). Some antidepressants, such as SSRIs and serotonin and norepinephrine reuptake inhibitors (SNRIs), are substrates of CYP2D6 but also inhibit CYP2D6, thereby reducing the creation of active tamoxifen metabolites and potentially its anticancer effect. Table 14 summarizes the inhibitory potential of different antidepressants on CYP2D6. Inhibitors can convert normal or ultrarapid metabolizers into intermediate or poor metabolizers. This is known as phenoconversion (*120*). Phenoconversion arises from drug-drug interactions, and when combined with the genetic background, it is referred to as drug-drug-gene interactions. Given its importance, the CPIC guidelines recommend considering CYP2D6 inhibitors when calculating the activity score (AS) for the CYP2D6 enzyme (*121*). Potent CYP2D6 inhibitors such as paroxetine and fluoxetine substantially reduce endoxifen concentrations in plasma (*122*). Weaker inhibitors like sertraline and citalopram may also reduce concentrations, but to a lesser extent (*123*). Based on this, the application of potent inhibitors should be avoided in patients with tamoxifen therapy (*124*). Nevertheless, the impact of weak-to-moderate inhibitors remains less clear, and interactions with other CYP enzymes involved in tamoxifen metabolism should also be considered (*125*).

**Table 14:** Antidepressants and their inhibitory effect on cytochrome P450 2D6

**Antidepressant**	CYP2D6 inhibition strength*	**Effect on endoxifen concentration^†^**	**Clinical recommendation**
Paroxetine, fluoxetine, bupropion	Strong	Markedly reduced	Avoid concomitant use with tamoxifen
Sertraline, citalopram, duloxetine	Moderate to weak	Slightly reduced	Use with caution and monitor clinical response
Venlafaxine, vortioxetine, escitalopram	Minimal or none	No relevant effect	Considered safer alternatives with tamoxifen
*Inhibition strength refers to the degree of cytochrome P450 2D6 enzyme inhibition, categorized according to *in vivo* effects on CYP2D6 metabolic activity and drug–drug interaction potential. ^†^Endoxifen is the main active metabolite of tamoxifen formed predominantly *via* CYP2D6-mediated metabolism; reduced CYP2D6 activity may lead to lower endoxifen plasma concentrations and potentially decreased therapeutic efficacy of tamoxifen. CYP2D6 - cytochrome P450 2D6.			

There has been much debate about whether antidepressant use affects breast cancer recurrence. Current evidence does not support the hypothesis that antidepressants worsen prognosis, although some data suggest a potential increased risk that requires further research (*126*).

According to CPIC and the DPWG, pharmacogenomic testing for *CYP2D6* is recommended to guide tamoxifen dosing, as well as to inform antidepressant prescribing (*121*, *127*). Considering the relevance of phenoconversion, CPIC guidelines also recommend including the effect of CYP2D6 inhibitors in activity score calculation. A recently developed CYP2D6 phenoconversion calculator allows manual prediction of CYP2D6 phenotype by integrating genotype, allele count, and concomitant medications, and is summarized in Table 15 (*128*). In conclusion, depression is a common condition among women with breast cancer receiving tamoxifen and should not be ignored. A conservative management approach emphasizes thoughtful choice of antidepressants to minimize the risk of clinically significant interactions, especially strong CYP2D6 inhibitors (paroxetine, fluoxetine, bupropion). Weak inhibitors, such as venlafaxine and vortioxetine, are generally safe to use with tamoxifen. Pharmacogenomics has an increasingly important role in optimizing therapy and improving outcomes in these patients.

**Table 15:** Cytochrome P450 2D6 phenoconversion calculator – Inputs and outputs

**Input required**	**Description**	**Output**
Genotype	Number and functional status of *CYP2D6* alleles	Base AS
Concomitant medications	List of co-administered CYP2D6 inhibitors	Adjusted AS
Final predicted phenotype	PM, IM, NM, UM (after considering inhibitors)	Guides dosing of tamoxifen and antidepressants dosing
Activity score (AS) represents a quantitative measure of CYP2D6 enzymatic function derived from the sum of allele activity values (*e.g.* 0 for no function, 0.5 for decreased function, 1 for normal function) (128). The adjusted AS accounts for phenoconversion due to concomitant CYP2D6 inhibitors, resulting in a modified predicted metabolizer phenotype. CYP2D6 - cytochrome P450 2D6. IM - intermediate metaboliser. NM - normal metaboliser. PM - poor metaboliser. UM - ultrarapid metaboliser.		

## Conclusion

Altogether, these advances confirm the position of laboratory diagnostics as the cornerstone of personalised medicine-bridging genetic discovery with clinical translation and transforming modern healthcare into a predictive, preventive, and truly patient-centred discipline.

## Data Availability

Not applicable.

## References

[1] Redekop WK Mladsi D. The faces of personalized medicine: a framework for understanding its meaning and scope. Value Health. 2013;16:S4–9. 10.1016/j.jval.2013.06.005 24034312

[2] Ginsburg GS Willard H. Genomic and personalized medicine: foundations and applications. Transl Res. 2009;154:277–87. 10.1016/j.trsl.2009.09.005 19931193

[3] Stenzinger A Moltzen EK Winkler E Molnar-Gabor F Malek N Costescu A Implementation of precision medicine in healthcare—A European perspective. J Intern Med. 2023;294:437–54. 10.1111/joim.13698 37455247

[4] Jørgensen JT. Twenty Years with Personalized Medicine: Past, Present, and Future of Individualized Pharmacotherapy. Oncologist. 2019;24:e432–40. 10.1634/theoncologist.2019-0054 30940745 PMC6656435

[5] Di Sanzo MD Cipolloni L Borro M Russa RL Santurro A Scopetti M Clinical Applications of Personalized Medicine: A New Paradigm and Challenge. Curr Pharm Biotechnol. 2017;18:194–203. 10.2174/1389201018666170224105600 28240172

[6] Chan IS Ginsburg GS. Personalized Medicine: Progress and Promise. Annu Rev Genomics Hum Genet. 2011;12:217–44. 10.1146/annurev-genom-082410-101446 21721939

[7] Cherny NI de Vries EGE Emanuel L Fallowfield L Francis PA Gabizon A Words Matter: Distinguishing “Personalized Medicine” and “Biologically Personalized Therapeutics”. J Natl Cancer Inst. 2014;106:dju321. 10.1093/jnci/dju321 25293984 PMC4568994

[8] Hora J Rambhia N Mani I. Drug repurposing for personalized medicine. Prog Mol Biol Transl Sci. 2024;207:107–22. 10.1016/bs.pmbts.2024.02.007 38942534

[9] Visvikis-Siest S Theodoridou D Kontoe MS Kumar S Marschler M. Milestones in Personalized Medicine: From the Ancient Time to Nowadays—the Provocation of COVID-19. Front Genet. 2020;11:569175. 10.3389/fgene.2020.569175 33424917 PMC7794000

[10] Watson JD Crick FHC. Molecular Structure of Nucleic Acids: A Structure for Deoxyribose Nucleic Acid. Nature. 1953;171:737–8. 10.1038/171737a0 13054692

[11] Franklin RE Gosling RG. Molecular Configuration in Sodium Thymonucleate. Nature. 1953;171:740–1. 10.1038/171740a0 13054694

[12] Ong T Ramsey BW. Cystic Fibrosis: A Review. JAMA. 2023;329:1859–71. 10.1001/jama.2023.8120 37278811

[13] Ratjen F Bell SC Rowe SM Goss CH Quittner AL Bush A. Cystic fibrosis. Nat Rev Dis Primers. 2015;1:15010. 10.1038/nrdp.2015.10 27189798 PMC7041544

[14] Burgel PR Durieu I Chiron R Ramel S Danner-Boucher I Prevotat A Rapid Improvement after Starting Elexacaftor-Tezacaftor-Ivacaftor in Patients with Cystic Fibrosis and Advanced Pulmonary Disease. Am J Respir Crit Care Med. 2021;204:64–73. 10.1164/rccm.202011-4153OC 33600738

[15] Daines CL Tullis E Costa S Linnemann RW Mall MA McKone EF Long-term safety and efficacy of elexacaftor/tezacaftor/ivacaftor in people with cystic fibrosis and at least one F508del allele: 144-week interim results from a 192-week open-label extension study. Eur Respir J. 2023;62:2202029. 10.1183/13993003.02029-2022 37945033 PMC10701091

[16] Fajac I Burgel PR Martin C. New drugs, new challenges in cystic fibrosis care. Eur Respir Rev. 2024;33:240045. 10.1183/16000617.0045-2024 39322262 PMC11423132

[17] Tješić-Drinković D Omerza L Bambir I Todorić I Aničić MN Senečić-Čala I Cistična fibroza – nove terapijske mogućnosti. Lijec Vjesn. 2022;144:27–35.

[18] Tješić-Drinković D Bambir I Tješić-Drinković D Vukić Dugac A Bambiar I Baretić M Cistična fibroza – ishodi liječenja u Hrvatskoj uz CFTR modulatore. Lijec Vjesn. 2023;145:121–30.

[19] Zolin A, Adamoli A, Bakkeheim E. ECFSPR Annual Report 2023. European Cystic Fibrosis Society [Internet]. [cited 2025 Oct 3]. Available from: https://www.ecfs.eu/projects/ecfs-patient-registry/annual-reports

[20] Hood L Flores M. A personal view on systems medicine and the emergence of proactive P4 medicine: predictive, preventive, personalized and participatory. N Biotechnol. 2012;29:613–24. 10.1016/j.nbt.2012.03.004 22450380

[21] Yu TW Kingsmore SF Green RC MacKenzie T Wasserstein M Caggana M Are we prepared to deliver gene-targeted therapies for rare diseases? Am J Med Genet C Semin Med Genet. 2023;193:7–12. 10.1002/ajmg.c.32029 36691939

[22] Groselj U Tansek MZ Battelino T. Fifty years of phenylketonuria newborn screening - A great success for many, but what about the rest? Mol Genet Metab. 2014;113:8–10. 10.1016/j.ymgme.2014.07.019 25174964

[23] Wilson JMG Jungner G. Principles and practice of screening for disease. J R Coll Gen Pract. 1968;16:318.4234760

[24] Andermann A Blancquaert I Beauchamp S Déry V. Revisiting Wilson and Jungner in the genomic age: a review of screening criteria over the past 40 years. Bull World Health Organ. 2008;86:317–9. 10.2471/BLT.07.050112 18438522 PMC2647421

[25] Schnabel-Besson E Mütze U Dikow N Hörster F Morath MA Alex K Wilson and Jungner Revisited: Are Screening Criteria Fit for the 21st Century? Int J Neonatal Screen. 2024;10:62. 10.3390/ijns10030062 39311364 PMC11417796

[26] Therrell BL Padilla CD Borrajo GJC Khneisser I Schielen PCJI Knight-Madden J Current Status of Newborn Bloodspot Screening Worldwide 2024: A Comprehensive Review of Recent Activities (2020-2023). Int J Neonatal Screen. 2024;10:38. 10.3390/ijns10020038 38920845 PMC11203842

[27] Hoffmann GF Lindner M Loeber JG. 50 years of newborn screening. J Inherit Metab Dis. 2014;37:163–4. 10.1007/s10545-014-9688-5 24526389

[28] Bhattacharjee A Sokolsky T Wyman SK Reese MG Puffenberger E Strauss K Development of DNA confirmatory and high-risk diagnostic testing for newborns using targeted next-generation DNA sequencing. Genet Med. 2015;17:337–47. 10.1038/gim.2014.117 25255367

[29] Šimić D Šarić A Škaričić A Lehman I Bunoza B Rako I One-Year Pilot Study Results of Newborn Screening for Spinal Muscular Atrophy in the Republic of Croatia. Int J Neonatal Screen. 2024;10:50. 10.3390/ijns10030050 39051406 PMC11270348

[30] Spiekerkoetter U Bick D Scott R Hopkins H Krones T Gross ES Genomic newborn screening: Are we entering a new era of screening? J Inherit Metab Dis. 2023;46:778–95. 10.1002/jimd.12650 37403863

[31] Betzler IR Hempel M Mütze U Kölker S Winkler E Dikow N Comparative analysis of gene and disease selection in genomic newborn screening studies. J Inherit Metab Dis. 2024;47:945–70. 10.1002/jimd.12750 38757337

[32] Rahimzadeh V Friedman JM de Wert G Knoppers BM. Exome/Genome-Wide Testing in Newborn Screening: A Proportionate Path Forward. Front Genet. 2022;13:865400. 10.3389/fgene.2022.865400 35860465 PMC9289115

[33] Gertsman I Barshop BA. Promises and pitfalls of untargeted metabolomics. J Inherit Metab Dis. 2018;41:355–66. 10.1007/s10545-017-0130-7 29536203 PMC5960440

[34] Huang X Wu D Zhu L Wang W Yang R Yang J Application of a next-generation sequencing (NGS) panel in newborn screening efficiently identifies inborn disorders of neonates. Orphanet J Rare Dis. 2022;17:66. 10.1186/s13023-022-02231-x 35193651 PMC8862216

[35] Downie L Bouffler SE Amor DJ Christodoulou J Yeung A Horton AE Gene selection for genomic newborn screening: Moving toward consensus? Genet Med. 2024;26:101077. 10.1016/j.gim.2024.101077 38275146

[36] Ashenden AJ Chowdhury A Anastasi LT Lam K Rozek T Ranieri E The Multi-Omic Approach to Newborn Screening: Opportunities and Challenges. Int J Neonatal Screen. 2024;10:42. 10.3390/ijns10030042 39051398 PMC11270328

[37] Prior TW Snyder PJ Rink BD Pearl DK Pyatt RE Mihal DC Newborn and carrier screening for spinal muscular atrophy. Am J Med Genet A. 2010;152A:1608–16. 10.1002/ajmg.a.33474 20578137

[38] D’Amico A Mercuri E Tiziano FD Bertini E. Spinal muscular atrophy. Orphanet J Rare Dis. 2011;6:71. 10.1186/1750-1172-6-71 22047105 PMC3231874

[39] Butchbach MER. Genomic Variability in the Survival Motor Neuron Genes (SMN1 and SMN2): Implications for Spinal Muscular Atrophy Phenotype and Therapeutics Development. Int J Mol Sci. 2021;22:7896. 10.3390/ijms22157896 34360669 PMC8348669

[40] Chaytow H Faller KME Huang YT Gillingwater TH. Spinal muscular atrophy: From approved therapies to future therapeutic targets for personalized medicine. Cell Rep Med. 2021;2:100346. 10.1016/j.xcrm.2021.100346 34337562 PMC8324491

[41] Farrar MA Kiernan MC. Spinal muscular atrophy - the dawning of a new era. Nat Rev Neurol. 2020;16:593–4. 10.1038/s41582-020-00410-7 32978515

[42] Farrar MA Park SB Vucic S Carey KA Turner BJ Gillingwater TH Emerging therapies and challenges in spinal muscular atrophy. Ann Neurol. 2017;81:355–68. 10.1002/ana.24864 28026041 PMC5396275

[43] Angilletta I Ferrante R Giansante R Lombardi L Babore A Dell’Elice A Spinal Muscular Atrophy: An Evolving Scenario through New Perspectives in Diagnosis and Advances in Therapies. Int J Mol Sci. 2023;24:14873. 10.3390/ijms241914873 37834320 PMC10573646

[44] Costa-Roger M Blasco-Pérez L Cuscó I Tizzano EF. The Importance of Digging into the Genetics of SMN Genes in the Therapeutic Scenario of Spinal Muscular Atrophy. Int J Mol Sci. 2021;22:9029. 10.3390/ijms22169029 34445733 PMC8396600

[45] De Vivo DC Bertini E Swoboda KJ Hwu WL Crawford TO Finkel RS NURTURE Study Group. Nusinersen initiated in infants during the presymptomatic stage of spinal muscular atrophy: Interim efficacy and safety results from the Phase 2 NURTURE study. Neuromuscul Disord. 2019;29:842–56. 10.1016/j.nmd.2019.09.007 31704158 PMC7127286

[46] Kölbel H Modler L Blaschek A Schara-Schmidt U Vill K Schwartz O Parental Burden and Quality of Life in 5q-SMA Diagnosed by Newborn Screening. Children (Basel). 2022;9:1829. 10.3390/children9121829 36553273 PMC9776462

[47] Erdos J Wild C. Mid- and long-term (at least 12 months) follow-up of patients with spinal muscular atrophy (SMA) treated with nusinersen, onasemnogene abeparvovec, risdiplam or combination therapies: A systematic review of real-world study data. Eur J Paediatr Neurol. 2022;39:1–10. 10.1016/j.ejpn.2022.04.006 35533607

[48] Walsh MF Chang VY Kohlmann WK Scott HS Cunniff C Bourdeaut F Recommendations for Childhood Cancer Screening and Surveillance in DNA Repair Disorders. Clin Cancer Res. 2017;23:e23–31. 10.1158/1078-0432.CCR-17-0465 28572264 PMC5697784

[49] Sharma R Lewis S Wlodarski MW. DNA Repair Syndromes and Cancer: Insights Into Genetics and Phenotype Patterns. Front Pediatr. 2020;8:570084. 10.3389/fped.2020.570084 33194896 PMC7644847

[50] Jahn A Rump A Widmann TJ Heining C Horak P Hutter B Comprehensive cancer predisposition testing within the prospective MASTER trial identifies hereditary cancer patients and supports treatment decisions for rare cancers. Ann Oncol. 2022;33:1186–99. 10.1016/j.annonc.2022.07.008 35988656

[51] Garber JE Offit K. Hereditary Cancer Predisposition Syndromes. J Clin Oncol. 2005;23:276–92. 10.1200/JCO.2005.10.042 15637391

[52] Imyanitov EN Kuligina ES Sokolenko AP Suspitsin EN Yanus GA Iyevleva AG Hereditary cancer syndromes. World J Clin Oncol. 2023;14:40–68. 10.5306/wjco.v14.i2.40 36908677 PMC9993141

[53] McDonnell JE Gild ML Clifton-Bligh RJ Robinson BG. Multiple endocrine neoplasia: an update. Intern Med J. 2019;49:954–61. 10.1111/imj.14394 31387156

[54] Stadler ZK Thom P Robson ME Weitzel JN Kauff ND Hurley KE Genome-Wide Association Studies of Cancer. J Clin Oncol. 2010;28:4255–67. 10.1200/JCO.2009.25.7816 20585100 PMC2953976

[55] Richards S Aziz N Bale S Bick D Das S Gastier-Foster J Standards and guidelines for the interpretation of sequence variants: a joint consensus recommendation of the American College of Medical Genetics and Genomics and the Association for Molecular Pathology. Genet Med. 2015;17:405–24. 10.1038/gim.2015.30 25741868 PMC4544753

[56] Plon SE Eccles DM Easton D Foulkes WD Genuardi M Greenblatt MS Sequence variant classification and reporting: recommendations for improving the interpretation of cancer susceptibility genetic test results. Hum Mutat. 2008;29:1282–91. 10.1002/humu.20880 18951446 PMC3075918

[57] Shendure J Ji H. Next-generation DNA sequencing. Nat Biotechnol. 2008;26:1135–45. 10.1038/nbt1486 18846087

[58] Levy SE Myers RM. Advancements in Next-Generation Sequencing. Annu Rev Genomics Hum Genet. 2016;17:95–115. 10.1146/annurev-genom-083115-022413 27362342

[59] Garutti M Foffano L Mazzeo R Michelotti A Da Ros L Viel A Hereditary Cancer Syndromes: A Comprehensive Review with a Visual Tool. Genes (Basel). 2023;14:1025. 10.3390/genes14051025 37239385 PMC10218093

[60] André T Shiu KK Kim TW Jensen BV Jensen LH Punt C Pembrolizumab in Microsatellite-Instability–High Advanced Colorectal Cancer. N Engl J Med. 2020;383:2207–18. 10.1056/NEJMoa2017699 33264544

[61] Robson M Im SA Senkus E Xu B Domchek SM Masuda N Olaparib for Metastatic Breast Cancer in Patients with a Germline BRCA Mutation. N Engl J Med. 2017;377:523–33. 10.1056/NEJMoa1706450 28578601

[62] Peltomäki P Nyström M Mecklin JP Seppälä TT. Lynch Syndrome Genetics and Clinical Implications. Gastroenterology. 2023;164:783–99. 10.1053/j.gastro.2022.08.058 36706841

[63] Rooney MM Miller KN Plichta JK. Genetics of Breast Cancer: Risk Models, Who to Test, and Management Options. Surg Clin North Am. 2023;103:35–47. 10.1016/j.suc.2022.08.016 36410352

[64] Schmidlen TJ Bristow SL Hatchell KE Esplin ED Nussbaum RL Haverfield EV. The Impact of Proband Indication for Genetic Testing on the Uptake of Cascade Testing Among Relatives. Front Genet. 2022;13:867226. 10.3389/fgene.2022.867226 35783293 PMC9243226

[65] Frey MK Ahsan MD Bergeron H Lin J Li X Fowlkes RK Cascade Testing for Hereditary Cancer Syndromes: Should We Move Toward Direct Relative Contact? A Systematic Review and Meta-Analysis. J Clin Oncol. 2022;40:4129–43. 10.1200/JCO.22.00303 35960887 PMC9746789

[66] Vogelstein B Papadopoulos N Velculescu VE Zhou S Diaz LA Jr Kinzler KW. Cancer genome landscapes. Science. 2013;339:1546–58. 10.1126/science.1235122 23539594 PMC3749880

[67] Garraway LA Lander ES. Lessons from the Cancer Genome. Cell. 2013;153:17–37. 10.1016/j.cell.2013.03.002 23540688

[68] Aaltonen LA Abascal F Abeshouse A Aburatani H Adams DJ Agrawal N Pan-cancer analysis of whole genomes. Nature. 2020;578:82–93. 10.1038/s41586-020-1969-6 32025007 PMC7025898

[69] Martincorena I Raine KM Gerstung M Dawson KJ Haase K Van Loo P Universal Patterns of Selection in Cancer and Somatic Tissues. Cell. 2017;171:1029–41.e21. 10.1016/j.cell.2017.09.042 29056346 PMC5720395

[70] Bányai L Trexler M Kerekes K Csuka O Patthy L. Use of signals of positive and negative selection to distinguish cancer genes and passenger genes. eLife. 2021;10:e59629. 10.7554/eLife.59629 33427197 PMC7877913

[71] Kumar S Warrell J Li S McGillivray PD Meyerson W Salichos L Passenger mutations in 2500 cancer genomes: Overall molecular functional impact and consequences. Cell. 2020;180:915–927.e16. 10.1016/j.cell.2020.01.032 32084333 PMC7210002

[72] PDQ® Cancer Genetics Editorial Board. PDQ Genetics of Breast and Gynecologic Cancers. Bethesda, MD: National Cancer Institute. Updated 03/06/2025. [Internet]. [cited 2025 Oct 15]. Available at: https://www.cancer.gov/types/breast/hp/breast-ovarian-genetics-pdq

[73] Gerlinger M Rowan AJ Horswell S Math M Larkin J Endesfelder D Intratumor heterogeneity and branched evolution revealed by multiregion sequencing. N Engl J Med. 2012;366:883–92. 10.1056/NEJMoa1113205 22397650 PMC4878653

[74] Jamal-Hanjani M Wilson GA McGranahan N Birkbak NJ Watkins TBK Veeriah S Tracking the Evolution of Non-Small-Cell Lung Cancer. N Engl J Med. 2017;376:2109–21. 10.1056/NEJMoa1616288 28445112

[75] Siravegna G Marsoni S Siena S Bardelli A. Integrating liquid biopsies into the management of cancer. Nat Rev Clin Oncol. 2017;14:531–48. 10.1038/nrclinonc.2017.14 28252003

[76] Goodwin S McPherson JD McCombie WR. Coming of age: ten years of next-generation sequencing technologies. Nat Rev Genet. 2016;17:333–51. 10.1038/nrg.2016.49 27184599 PMC10373632

[77] Konnick EQ Pritchard CC. Germline, hematopoietic, mosaic, and somatic variation: interplay between inherited and acquired genetic alterations in disease assessment. Genome Med. 2016;8:100. 10.1186/s13073-016-0350-8 27716394 PMC5050638

[78] Alexandrov LB Nik-Zainal S Wedge DC Aparicio SAJR Behjati S Biankin AV Signatures of mutational processes in human cancer. Nature. 2013;500:415–21. 10.1038/nature12477 23945592 PMC3776390

[79] Strickler JH Hanks BA Khasraw M. Tumor Mutational Burden as a Predictor of Immunotherapy Response: Is More Always Better? Clin Cancer Res. 2021;27:1236–41. 10.1158/1078-0432.CCR-20-3054 33199494 PMC9912042

[80] Smith CEP Prasad V. Targeted Cancer Therapies. Am Fam Physician. 2021;103:155–63. 33507053

[81] Yin J Shen S Shi Q. Challenges, opportunities, and innovative statistical designs for precision oncology trials. Ann Transl Med. 2022;10:1038. 10.21037/atm-22-356 36267789 PMC9577796

[82] Moore DC Guinigundo AS. Biomarker-Driven Oncology Clinical Trials: Novel Designs in the Era of Precision Medicine. J Adv Pract Oncol. 2023;14:9–13. 10.6004/jadpro.2023.14.3.16 37206904 PMC10190802

[83] Fountzilas E Tsimberidou AM Vo HH Kurzrock R. Clinical trial design in the era of precision medicine. Genome Med. 2022;14:101. 10.1186/s13073-022-01102-1 36045401 PMC9428375

[84] Meyer UA Zanger UM. Molecular mechanisms of genetic polymorphisms of drug metabolism. Annu Rev Pharmacol Toxicol. 1997;37:269–96. 10.1146/annurev.pharmtox.37.1.269 9131254

[85] Roden DM McLeod HL Relling MV Williams MS Mensah GA Peterson JF Pharmacogenomics. Lancet. 2019;394(10197):521–32. 10.1016/S0140-6736(19)31276-0 31395440 PMC6707519

[86] Pirmohamed M. Pharmacogenomics: current status and future perspectives. Nat Rev Genet. 2023;24:350–62. 10.1038/s41576-022-00572-8 36707729

[87] Sadee W Wang D Hartmann K Toland AE. Pharmacogenomics: Driving Personalized Medicine. Pharmacol Rev. 2023;75:789–814. 10.1124/pharmrev.122.000810 36927888 PMC10289244

[88] Caudle KE, Whirl-Carrillo M, Relling MV, Hoffman JM, Donnelly RS, Haidar CE, et al. Advancing Clinical Pharmacogenomics Worldwide Through the Clinical Pharmacogenetics Implementation Consortium (CPIC). Clin Pharmacol Ther. 2025 Jul 18 [cited 2025 Oct7] [Epub ahead of print]. 10.1002/cpt.70005 PMC13037831 40678821

[89] Haidar CE Crews KR Hoffman JM Relling MV Caudle KE. Advancing Pharmacogenomics from Single-Gene to Preemptive Testing. Annu Rev Genomics Hum Genet. 2022;23:449–73. 10.1146/annurev-genom-111621-102737 35537468 PMC9483991

[90] Kabbani D Akika R Wahid A Daly AK Cascorbi I Zgheib NK. Pharmacogenomics in practice: a review and implementation guide. Front Pharmacol. 2023;14:1189976. 10.3389/fphar.2023.1189976 37274118 PMC10233068

[91] Swen JJ van der Wouden CH Manson LE Abdullah-Koolmees H Blagec K Blagus T A 12-gene pharmacogenetic panel to prevent adverse drug reactions: an open-label, multicentre, controlled, cluster-randomised crossover implementation study. Lancet. 2023;401:347–56. 10.1016/S0140-6736(22)01841-4 36739136

[92] Lauschke VM Zhou Y Ingelman-Sundberg M. Pharmacogenomics Beyond Single Common Genetic Variants: The Way Forward. Annu Rev Pharmacol Toxicol. 2024;64:33–51. 10.1146/annurev-pharmtox-051921-091209 37506333

[93] Weinshilboum RM Wang L. Pharmacogenomics: Precision Medicine and Drug Response. Mayo Clin Proc. 2017;92:1711–22. 10.1016/j.mayocp.2017.09.001 29101939 PMC5682947

[94] Tayeh MK Gaedigk A Goetz MP Klein TE Lyon E McMillin GA Clinical pharmacogenomic testing and reporting: A technical standard of the American College of Medical Genetics and Genomics (ACMG). Genet Med. 2022;24:759–68. 10.1016/j.gim.2021.12.009 35177334

[95] Swen JJ Nijenhuis M de Boer A Grandia L Maitland-van der Zee AH Mulder H Pharmacogenetics: from bench to byte--an update of guidelines. Clin Pharmacol Ther. 2011;89:662–73. 10.1038/clpt.2011.34 21412232

[96] Thorn CF Klein TE Altman RB. PharmGKB: the Pharmacogenomics Knowledge Base. Methods Mol Biol. 2013;1015:311–20. 10.1007/978-1-62703-435-7_20 23824865 PMC4084821

[97] Bozina N Bradamante V Lovrić M. Genetic polymorphism of metabolic enzymes P450 (CYP) as a susceptibility factor for drug response, toxicity, and cancer risk. Arh Hig Rada Toksikol. 2009;60:217–42. 10.2478/10004-1254-60-2009-1885 19581216

[98] Rendic SP Guengerich FP. Human Family 1–4 cytochrome P450 enzymes involved in the metabolic activation of xenobiotic and physiological chemicals: an update. Arch Toxicol. 2021;95:395–472. 10.1007/s00204-020-02971-4 33459808 PMC7880545

[99] Caudle KE Dunnenberger HM Freimuth RR Peterson JF Burlison JD Whirl-Carrillo M Standardizing terms for clinical pharmacogenetic test results: consensus terms from the Clinical Pharmacogenetics Implementation Consortium (CPIC). Genet Med. 2017;19:215–23. 10.1038/gim.2016.87 27441996 PMC5253119

[100] Cooper-DeHoff RM Niemi M Ramsey LB Luzum JA Tarkiainen EK Straka RJ The Clinical Pharmacogenetics Implementation Consortium Guideline for SLCO1B1, ABCG2, and CYP2C9 genotypes and Statin-Associated Musculoskeletal Symptoms. Clin Pharmacol Ther. 2022;111:1007–21. 10.1002/cpt.2557 35152405 PMC9035072

[101] Litonius K Kulla N Falkenbach P Kristiansson K Tarkiainen EK Ukkola-Vuoti L Value of Pharmacogenetic Testing Assessed with Real-World Drug Utilization and Genotype Data. Clin Pharmacol Ther. 2025;117:278–88. 10.1002/cpt.3458 39365028 PMC11652815

[102] Božina N Vrkić Kirhmajer M Šimičević L Ganoci L Mirošević Skvrce N Klarica Domjanović I Use of pharmacogenomics in elderly patients treated for cardiovascular diseases. Croat Med J. 2020;61:147–58. 10.3325/cmj.2020.61.147 32378381 PMC7230415

[103] Cheng CM So TW Bubp JL. Characterization of Pharmacogenetic Information in Food and Drug Administration Drug Labeling and the Table of Pharmacogenetic Associations. Ann Pharmacother. 2021;55:1185–94. 10.1177/1060028020983049 33384014

[104] Shekhani R Steinacher L Swen JJ Ingelman-Sundberg M. Evaluation of Current Regulation and Guidelines of Pharmacogenomic Drug Labels: Opportunities for Improvements. Clin Pharmacol Ther. 2020;107:1240–55. 10.1002/cpt.1720 31715018 PMC7232863

[105] Deb S Hopefl R Reeves AA Cvetkovic D. ADME Gene-Related Pharmacogenomic Labeling of FDA-Approved Drugs: Comparison with Clinical Pharmacogenetics Implementation Consortium (CPIC) Evidence Levels. Medicines (Basel). 2024;11:6. 10.3390/medicines11030006 38535119 PMC10972161

[106] Zhou Y Lauschke VM. Population pharmacogenomics: an update on ethnogeographic differences and opportunities for precision public health. Hum Genet. 2022;141:1113–36. 10.1007/s00439-021-02385-x 34652573 PMC9177500

[107] Pilevarzadeh M Amirshahi M Afsargharehbagh R Rafiemanesh H Hashemi SM Balouchi A. Global prevalence of depression among breast cancer patients: a systematic review and meta-analysis. Breast Cancer Res Treat. 2019;176:519–33. 10.1007/s10549-019-05271-3 31087199

[108] Howlader N Chen VW Ries LAG Loch MM Lee R DeSantis C Overview of breast cancer collaborative stage data items--their definitions, quality, usage, and clinical implications: a review of SEER data for 2004-2010. Cancer. 2014;120:3771–80. 10.1002/cncr.29059 25412389

[109] Zembutsu H Nakamura S Akashi-Tanaka S Kuwayama T Watanabe C Takamaru T Significant Effect of Polymorphisms in CYP2D6 on Response to Tamoxifen Therapy for Breast Cancer: A Prospective Multicenter Study. Clin Cancer Res. 2017;23:2019–26. 10.1158/1078-0432.CCR-16-1779 27797974

[110] Kiyotani K Mushiroda T Imamura CK Hosono N Tsunoda T Kubo M Significant Effect of Polymorphisms in CYP2D6 and ABCC2 on Clinical Outcomes of Adjuvant Tamoxifen Therapy for Breast Cancer Patients. J Clin Oncol. 2010;28:1287–93. 10.1200/JCO.2009.25.7246 20124171 PMC4872305

[111] Gaedigk A Simon SD Pearce RE Bradford LD Kennedy MJ Leeder JS. The CYP2D6 activity score: translating genotype information into a qualitative measure of phenotype. Clin Pharmacol Ther. 2008;83:234–42. 10.1038/sj.clpt.6100406 17971818

[112] Ji Y Skierka JM Blommel JH Moore BE VanCuyk DL Bruflat JK Preemptive Pharmacogenomic Testing for Precision Medicine: A Comprehensive Analysis of Five Actionable Pharmacogenomic Genes Using Next-Generation DNA Sequencing and a Customized CYP2D6 Genotyping Cascade. J Mol Diagn JMD. 2016;18:438–45. 10.1016/j.jmoldx.2016.01.003 26947514 PMC4851731

[113] Nishida Y Fukuda T Yamamoto I Azuma J. CYP2D6 genotypes in a Japanese population: low frequencies of CYP2D6 gene duplication but high frequency of CYP2D6*10. Pharmacogenetics. 2000;10:567–70. 10.1097/00008571-200008000-00010 10975611

[114] Bradford LD. CYP2D6 allele frequency in European Caucasians, Asians, Africans and their descendants. Pharmacogenomics. 2002;3:229–43. 10.1517/14622416.3.2.229 11972444

[115] Lu J Li H Guo P Shen R Luo Y Ge Q The effect of CYP2D6 *10 polymorphism on adjuvant tamoxifen in Asian breast cancer patients: a meta-analysis. Onco Targets Ther. 2017;10:5429–37. 10.2147/OTT.S149197 29180876 PMC5692201

[116] Schroth W Antoniadou L Fritz P Schwab M Muerdter T Zanger UM Breast Cancer Treatment Outcome With Adjuvant Tamoxifen Relative to Patient CYP2D6 and CYP2C19 Genotypes. J Clin Oncol. 2007;25:5187–93. 10.1200/JCO.2007.12.2705 18024866

[117] Mayer SE Weiss NS Chubak J Doody DR Carlson CS Makar KW CYP2D6-inhibiting medication use and inherited CYP2D6 variation in relation to adverse breast cancer outcomes after tamoxifen therapy. Cancer Causes Control. 2019;30:103–12. 10.1007/s10552-018-1117-x 30542984 PMC6353670

[118] Stearns V Johnson MD Rae JM Morocho A Novielli A Bhargava P Active tamoxifen metabolite plasma concentrations after coadministration of tamoxifen and the selective serotonin reuptake inhibitor paroxetine. J Natl Cancer Inst. 2003;95:1758–64. 10.1093/jnci/djg108 14652237

[119] Schroth W Winter S Mürdter T Schaeffeler E Eccles D Eccles B Improved Prediction of Endoxifen Metabolism by CYP2D6 Genotype in Breast Cancer Patients Treated with Tamoxifen. Front Pharmacol. 2017;8:582. 10.3389/fphar.2017.00582 28955222 PMC5609540

[120] Nahid NA Johnson JA. CYP2D6 pharmacogenetics and phenoconversion in personalized medicine. Expert Opin Drug Metab Toxicol. 2022;18:769–85. 10.1080/17425255.2022.2160317 36597259 PMC9891304

[121] Goetz MP Sangkuhl K Guchelaar HJ Schwab M Province M Whirl-Carrillo M Clinical Pharmacogenetics Implementation Consortium (CPIC) Guideline for CYP2D6 and Tamoxifen Therapy. Clin Pharmacol Ther. 2018;103:770–7. 10.1002/cpt.1007 29385237 PMC5931215

[122] Jin Y Desta Z Stearns V Ward B Ho H Lee KH CYP2D6 genotype, antidepressant use, and tamoxifen metabolism during adjuvant breast cancer treatment. J Natl Cancer Inst. 2005;97:30–9. 10.1093/jnci/dji005 15632378

[123] Borges S Desta Z Li L Skaar TC Ward BA Nguyen A Quantitative effect of CYP2D6 genotype and inhibitors on tamoxifen metabolism: implication for optimization of breast cancer treatment. Clin Pharmacol Ther. 2006;80:61–74. 10.1016/j.clpt.2006.03.013 16815318

[124] Sideras K Ingle JN Ames MM Loprinzi CL Mrazek DP Black JL Coprescription of Tamoxifen and Medications That Inhibit CYP2D6. J Clin Oncol. 2010;28:2768–76. 10.1200/JCO.2009.23.8931 20439629 PMC2881853

[125] Safgren SL Suman VJ Leon Ferre RA Kosel ML Stearns V Henry NL The impact of coadministration of venlafaxine, citalopram or gabapentin on the metabolic activation of tamoxifen. Breast Cancer Res Treat. 2025;211:261–70. 10.1007/s10549-025-07644-3 40011368 PMC12173219

[126] Chubak J Bowles EJA Yu O Buist DSM Fujii M Boudreau DM. Breast cancer recurrence in relation to antidepressant use. Cancer Causes Control. 2016;27:125–36. 10.1007/s10552-015-0689-y 26518198 PMC4913547

[127] ClinPGx [Internet]. [cited 2025 Oct 15]. The Royal Dutch Pharmacists Association - Pharmacogenetics Working Group. Annotation of DPWG Guideline for tamoxifen and CYP2D6. November 2018 Update. Available from: https://www.clinpgx.org/guidelineAnnotation/PA166104966/annotation

[128] CYP2D6 Phenoconversion Calculator. Precision Medicine Program. UF Health. University of Florida [Internet]. [cited 2025 Oct 15]. Available from: https://precisionmedicine.ufhealth.org/how-to-interpret-results/phenoconversion-calculator/.

